# Sleep as a Developmental Process: A Systematic Review of Cognitive, Emotional, and Behavioral Outcomes in Children Aged 6–12 Years

**DOI:** 10.3390/clockssleep7040066

**Published:** 2025-11-14

**Authors:** Adriana Félix, Adelinda Candeias

**Affiliations:** School of Health and Human Development, Comprehensive Health Research Centre, Universidade de Évora, 7000-812 Évora, Portugal; d60733@alunos.uevora.pt

**Keywords:** sleep, child development, sleep duration, sleep quality, cognitive outcomes, emotional outcomes, behavioral problems, physical health, screen time, digital media

## Abstract

Sleep is essential for child development, influencing cognition, emotional regulation, behavior, and physical health. Recent studies increasingly frame sleep as both a key developmental process and a modifiable factor shaped by, and shaping environmental risks—including digital screen exposure and psychosocial stress. This systematic review synthesized empirical findings from cross-sectional and cohort studies published between 2019 and 2024 on the associations between sleep duration, quality, and patterns and developmental outcomes in typically developing children aged 6–12 years. Searches were conducted in EBSCO, Scopus, and Web of Science databases, yielding 99 records, of which 20 met inclusion criteria. Methodological quality was evaluated using Joanna Briggs Institute tools. Findings show consistent associations between better sleep and enhanced cognitive performance, emotional well-being, and reduced behavioral problems. Some studies identified sleep as a mediator between screen use and behavioral difficulties, whit additional moderating effects related to gender and socioeconomic status. However, most studies used cross-sectional designs and self-reported measures, limiting causal interpretation. Overall, sleep emerge as a potentially modifiable factor influencing developmental outcomes, based on correlational evidence. Future research should prioritize longitudinal and ecologically valid designs, objective measures, and computational approaches to identify sleep-related risk profiles and guide early interventions.

## 1. Introduction

Sleep is a foundational biological process that plays a pivotal role in children’s physical maturation, neurocognitive development, emotional regulation, and behavioral adjustment. During middle childhood—a developmental stage marked by increased environmental demands and the consolidation of executive functions—sleep is increasingly recognized as a dynamic system integrating physiological, psychological, and contextual inputs [1,2].

Across this stage, transformations in sleep architecture, duration, and quality are not merely developmental by-products but active contributors to neural plasticity and cognitive functioning. Adequate sleep has been consistently associated with enhanced memory consolidation, attentional control, and emotional resilience, as demonstrated in both neurotypical populations and in children with learning difficulties [3,4,5]. Conversely, chronic sleep disturbances—arising from reduced duration, poor quality, or inconsistencies—are associated with impaired learning processes, emotional dysregulation, and atypical neurodevelopmental trajectories [6,7]. Recent neuroimaging shows that such disruptions can impair connectivity in networks critical to executive functioning and emotional function, particularly the prefrontal cortex and limbic system [1,8].

Although these links have been emphasized in clinical populations, such as children with Autism Spectrum Disorder or ADHD, growing evidence shows that even typically developing children face similar risks [9,10,11]. This reflects profound shift in modern childhood contexts, including variations in parenting, socioeconomic disparities, and especially increased exposure to digital Technologies, which collectively modulate sleep behaviors and their developmental outcomes [12,13].

Large-scale and longitudinal research demonstrates that sleep duration and quality broadly affect cognitive, behavioral, and physical well-being in typically developing children. Shorter or fragmented sleep is tied to diminished attention, memory, and academic achievement. Sleep disturbances are common, correlating with poorer social functioning and greater emotional difficulties even among healthy school-aged children. While problems are more severe in neurodevelopmental disorders, similar (if less marked) patterns now warrant attention in neurotypical populations, emphasizing the potential for prevention and early intervention [14,15,16].

Among environmental factors, digital screen exposure has emerged as a particularly salient. Screen time can exacerbate behavioral and emotional problems via delayed sleep onset, reduced sleep efficiency, and physiological arousal [17,18]. Still, most evidence remains correlational. Robust longitudinal studies are needed to clarify causal directions. The possibility that sleep mediates or moderates the effects of environmental pressures demands further study, especially given the cross-sectional nature of most available data.

Past reviews have primarily focused on clinical samples or on sleep as an outcome. Fewer have systematically reviewed sleep’s developmental functions or synthesized evidence spanning both environmental exposures and everyday lifestyle factors in typically developing children.

Accordingly, this systematic review synthesizes recent (2019–2024) evidence on the links between sleep and cognitive, emotional, and behavioral outcomes in children aged 6 to 12 years. More than mapping associations, the review considers developmental mechanisms and life context—such as digital media—through which sleep shapes trajectories of psychological functioning. It also identifies conditions where sleep may buffer or exacerbate vulnerabilities, offering leverage points for future research and preventive action.

## 2. Methods

### 2.1. Systematic Review Registration

This systematic review was conducted in accordance with the PRISMA 2020 guidelines. The review was not prospectively registered in any registry, as it was developed as part of a broader research project on developmental mechanisms of sleep. However, all methodological decisions—including inclusion criteria, databases searched, and analytic procedures—were defined a priori and documented to ensure transparency and reproducibility.

### 2.2. Information Sources and Search Strategy

The information sources for this systematic review included three major academic databases: EBSCO, Scopus, and Web of Science. The search was conducted on **13 December 2024**, to ensure the inclusion of the most recent publications. The full search strategies (including exact terms and Boolean operators for each database) are provided in Supplementary Material S1, in accordance with PRISMA guidelines to ensure reproducibility. These databases were selected to ensure broad coverage of biomedical, educational, and psychological research relevant to child development, and represent major sources for peer-reviewed literature in this field. The search was conducted on 13 December 2024, to ensure the inclusion of the most recent publications.

The search strategy was tailored for each database, using a combination of keywords and Boolean operators to capture relevant studies. The primary search terms included combinations of “sleep patterns,” “sleep quality,” “sleep duration,” “neurodevelopment,” “cognitive development,” “emotional development,” “children,” and “childhood.” Specific search strings were adapted for each database to align with their unique indexing and syntax rules. Full details for each database are available in Supplementary Material S1.

For EBSCO, the search query focused on titles and subject terms, combining: TI (sleep patterns OR sleep quality OR sleep duration) AND SU (neurodevelopment OR cognitive development OR emotional development) AND (children OR childhood). For Scopus, the search was performed in titles, abstracts, and keywords using the following string: TITLE-ABS-KEY (sleep AND patterns OR sleep AND quality OR sleep AND duration) AND TITLE-ABS-KEY (neurodevelopment OR cognitive AND development OR emotional AND development) AND TITLE-ABS-KEY (children OR childhood) AND PUBYEAR > 2018 AND PUBYEAR < 2025 AND (LIMIT-TO (LANGUAGE, “English”)) AND (LIMIT-TO (PUBSTAGE, “final”)). In Web of Science, the search targeted titles and topics with the following terms: (sleep patterns OR sleep quality OR sleep duration) (Title) AND (neurodevelopment OR cognitive development OR emotional development) (Topic) AND (children OR childhood) (Topic) AND (2019 OR 2020 OR 2021 OR 2022 OR 2023 OR 2024) (Publication Years) AND English (Languages).

All search results were exported, and duplicates were removed before the screening process began.

### 2.3. Eligibility Criteria

The eligibility criteria for this systematic review were carefully defined to ensure the inclusion of studies that directly address the research question and maintain methodological rigor. The inclusion criteria specified that studies must focus on typically developing children aged 6–12 years and employ empirical research designs, including quantitative, qualitative, or mixed-methods approaches, with primary data collection and analysis. This age range was chosen because it represents a critical developmental period when children are typically enrolled in formal schooling. During this stage, sleep patterns are known to significantly impact cognitive, emotional, and neurodevelopmental outcomes, which are essential for academic performance and social functioning. Limiting the age range excludes younger children, whose developmental needs and sleep patterns may differ due to rapid physiological changes, and adolescents, who experience distinct sleep-related challenges influenced by puberty.

Only articles published in English between 2019 and 2024 were considered, and studies had to specifically examine the relationship between sleep patterns, sleep quality, or sleep duration and neurodevelopmental, cognitive, or emotional outcomes in children. Furthermore, only full-text articles available for review were included.

Studies were excluded if they included data outside the target age range of 6–12 years unless stratified data were available for the target group. Additionally, studies focusing on children diagnosed with specific clinical or neurodevelopmental disorders, such as Attention-Deficit/Hyperactivity Disorder (ADHD), Autism Spectrum Disorder (ASD), epilepsy, congenital heart diseases, or other chronic medical conditions, were excluded. This exclusion was necessary to avoid confounding effects, as such conditions can independently influence sleep patterns and neurodevelopmental outcomes, introducing variability that might obscure generalizable insights applicable to typically developing children.

Other exclusions included review articles, editorials, opinion pieces, conference abstracts, dissertations, non-English publications, and articles duplicated across multiple databases. Studies lacking sufficient methodological details or outcome data were also excluded. These criteria were systematically applied across all selected databases (EBSCO, Scopus, Web of Science https://www.webofscience.com/wos/woscc/summary/629ba9e5-0a47-4371-8b8b-880d9b14a373-01368f2d95/relevance/1 (accessed on 13 December 2024)) to ensure consistency and transparency in the study selection process. Studies providing aggregated data for age ranges exceeding 6–12 years were excluded unless the majority of participants or stratified data fit the target range.

### 2.4. Study Selection

The study selection process followed the PRISMA 2020 guidelines to ensure transparency and methodological rigor. After completing the search across the selected databases (EBSCO, Scopus, and Web of Science) and removing duplicate records, the screening process was conducted in two distinct stages: title and abstract screening, followed by full-text review. In the first stage, the titles and abstracts of all retrieved articles were screened to assess their relevance based on the predefined eligibility criteria. Articles that did not meet the inclusion criteria or explicitly met exclusion criteria (e.g., studies focusing on children with specific disorders such as ADHD, autism, or epilepsy) were excluded at this stage. In the second stage, the full texts of the remaining articles were reviewed to confirm their eligibility for inclusion. Any uncertainties during the screening process were resolved through careful consideration of the predefined criteria. The entire study selection process is summarized and visually represented in a PRISMA flow diagram, which will be included in the Results section. This diagram details the number of records identified, screened, excluded, and included, providing a transparent overview of the study selection workflow.

A total of 99 records were identified from three databases: EBSCO, Scopus, and Web of Science. After removing 21 duplicate records, 78 records remained for screening. During the initial screening of titles and abstracts, 32 records were excluded due to lack of full text (19), being literature reviews (7), or addressing specific problematics that fell outside the scope of this review (6). This left 46 articles for full-text assessment. Of these, 26 were excluded because they involved a non-matching target population, leaving 20 studies that met all inclusion criteria. These selected studies were included in the final synthesis.

**Figure 1:**
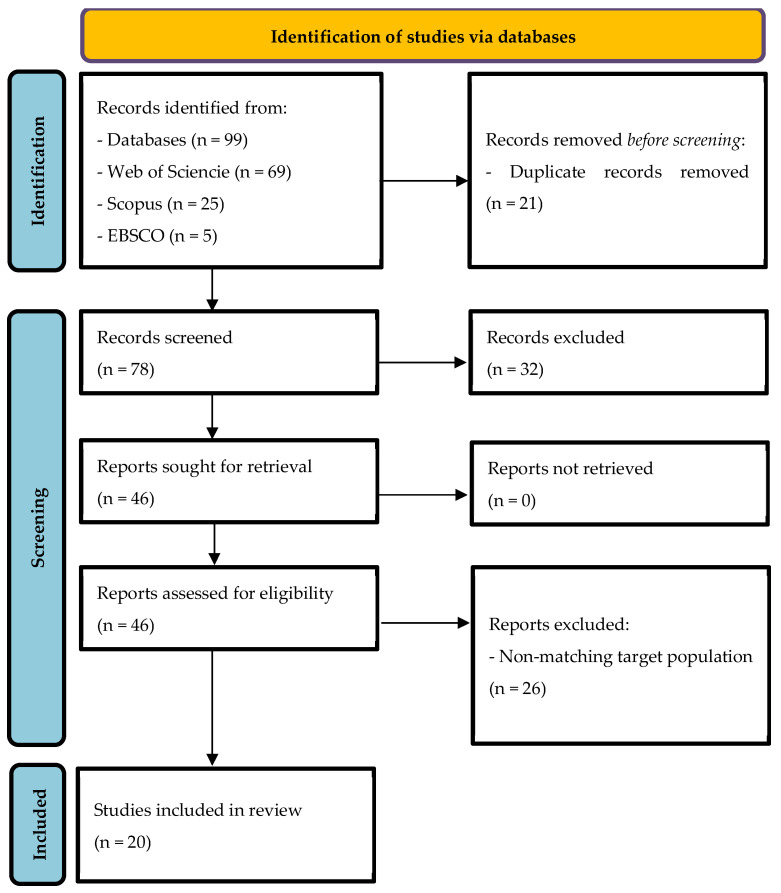
PRISMA Flow Diagram of Included studies.

The study selection process is visually summarized in the PRISMA 2020 flow diagram (Figure 1), which details the number of records identified, screened, excluded, and included.

### 2.5. Data Extraction

The data extraction process was conducted systematically to ensure accuracy and consistency. A standardized data extraction template was developed and used in Microsoft Excel to record relevant information from each included study. The variables extracted included the following:

Study characteristics: author(s), year of publication, title, journal, and study location.

Study design: type of study (e.g., cross-sectional, longitudinal), methods, and duration of follow-up (if applicable).

Sample characteristics: sample size, age range, gender distribution, and any specific inclusion or exclusion criteria.

Outcomes: primary and secondary outcomes measured, including sleep duration, quality, and any associated variables such as cognitive function, behavior, or health outcomes.

Key findings: main results, including effect sizes, associations, and significant conclusions.

Limitations: study-specific limitations as reported by the authors.

Two independent reviewers (Author 1 and Author 2) performed the screening of titles, abstracts, and full texts. Any uncertainties during the extraction process were resolved through careful review and verification of the original source material. Data extraction was also independently performed by both reviewers using standardized forms based on PRISMA 2020 guidelines. Consensus was reached on all final inclusions and extracted data through iterative comparison. This systematic approach ensured a comprehensive and reliable dataset for the subsequent analysis.

### 2.6. Risk of Bias Assessment

The risk of bias in the included studies was assessed using the Joanna Briggs Institute (JBI) tools designed for observational and cohort studies, as outlined in the JBI Manual for Evidence Synthesis [16]. Each study was evaluated for methodological rigor and potential sources of bias based on the specific criteria in the relevant JBI checklists.

For observational studies, the assessment criteria included clarity of inclusion criteria, detailed descriptions of study settings and participants, the validity and reliability of exposure and outcome measurements, the identification and management of confounding factors, and the appropriateness of statistical analyses. Cohort studies were further evaluated for the comparability of study groups, the completeness of follow-up, and the adequacy of follow-up duration.

Most included studies demonstrated robust methodologies with well-defined inclusion criteria, valid measurements, and appropriate statistical approaches. However, some studies reported limited strategies for addressing confounding factors, and a subset of cohort studies lacked sufficient follow-up descriptions. These limitations were considered in the interpretation of the findings and their implications for the broader literature.

Overall, the risk of bias across the studies was judged to be low to moderate, with most studies demonstrating adequate methodological rigor to support their conclusions.

## 3. Results

### 3.1. Quality of the Primary Studies

Regarding the quality of the methodological aspects of the primary studies we identified that 80% of the studies presented a high quality and 20% presented a reasonable quality (Table 1 and Table 2).

### 3.2. Study Characteristics

**Figure 2:**
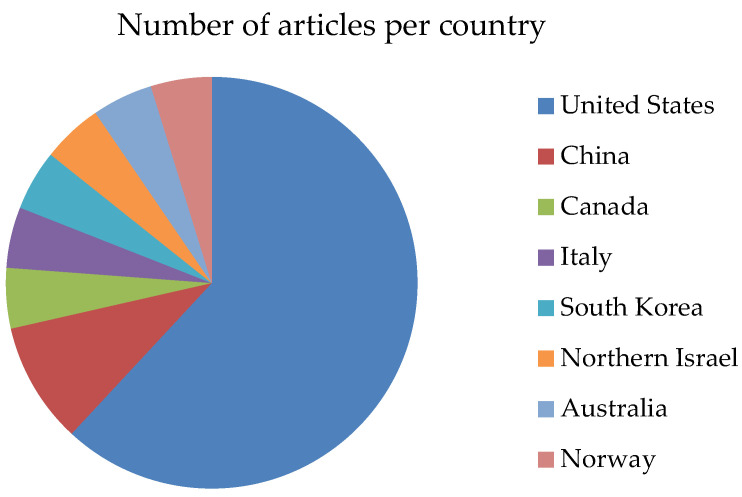
Number of articles per country.

**Figure 3:**
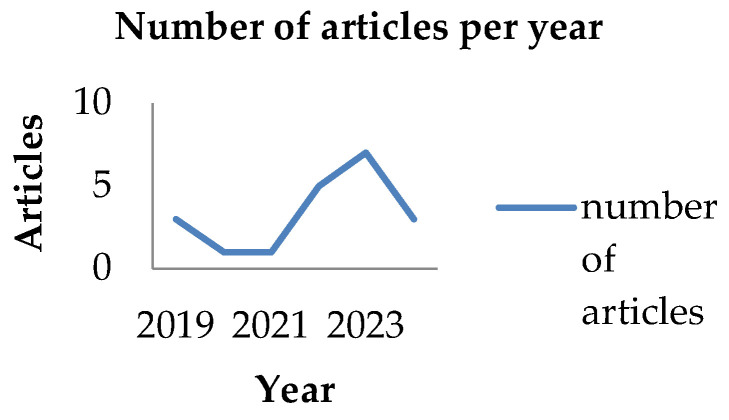
Number of articles per year.

The included studies were diverse in design, population, and outcomes, reflecting the multifaceted relationship between sleep and its effects on cognitive, behavioral, and emotional development in children aged 6–12 years. The studies were conducted across a variety of geographic locations, with the majority originating from the United States (65%), followed by China, Canada, Italy, South Korea, Northern Israel, Australia, and Norway. In terms of publication trends, the studies were distributed over six years, with three articles published in 2019, one in 2020, one in 2021, five in 2022, seven in 2023, and three in 2024, highlighting an increasing research focus on sleep and neurodevelopmental outcomes in children during this period. The lower number of articles in 2020 and 2021 may reflect the widespread disruptions caused by the COVID-19 pandemic, which significantly impacted research activities and publication timelines across various fields. (see Figure 2 and Figure 3).

The selected studies reflect a broad exploration of the relationship between sleep and various developmental outcomes in children aged 6–12 years. These studies encompass diverse geographic locations, methodologies, and populations, offering valuable insights into cognitive, emotional, and behavioral aspects of child development. Table 3 summarizes the study characteristics, including their objectives, designs, population focus, outcome measures, and key findings. The inclusion of general populations, as well as specific subgroups such as children with high screen time exposure, low physical activity levels, or those in foster care, highlights the complexity of factors influencing sleep-related outcomes. This diversity provides a comprehensive foundation for understanding the multifaceted nature of sleep’s role in childhood development.

Table 4 provides a detailed summary of each study’s objectives, design, outcome measures, results, and limitations. The outcomes assessed were equally diverse, ranging from cognitive performance and emotional well-being to behavioral issues and physical health metrics like body mass index (BMI). These findings underline the complex and interconnected roles of sleep across various domains of child development.

### 3.3. Thematic Synthesis of Findings

The findings from the included studies collectively reinforce the multifactorial role of sleep in childhood development, with consistent associations across cognitive performance, behavioral outcomes, emotional well-being, and physical health. More than isolated effects, sleep emerges as a dynamic variable that both reflects and shapes developmental trajectories through its interaction with environmental and individual factors.

#### 3.3.1. Cognitive Performance

A number of studies confirmed that sleep duration and quality are key predictors of cognitive functioning and neurodevelopmental integrity.

Lee et al. [11] found that boys sleeping ≥ 10 h per night had a mean IQ score that was 10.5 points higher (95% CI 2.7–18.4, *p* = 0.009) compared to boys sleeping less than 8 h. The same pattern was observed for verbal IQ (*p* = 0.041) and performance IQ (*p* = 0.023). No significant associations were found in girls. Additionally, longer sleep duration was associated with an increase in verbal IQ measures (β = 0.55, *p* = 0.030) across the sample, confirming a dose-dependent relationship between sleep duration and IQ specifically for boys.

Yan et al. [35] demonstrated that each additional hour of sleep duration correlated positively with frontoparietal activation during working memory tasks, particularly among girls (β = 0.03, *p* = 0.008).

Cheng et al. [10] reported that shorter sleep duration was associated with reduced gray matter volume in the prefrontal cortex, and medial orbitofrontal cortex (β range: 0.05–0.09, *p* < 0.001). Lower sleep duration also corresponded to decreased cognitive scores (β = 0.03, *p* < 0.001) and reduced functional connectivity between executive regions.

#### 3.3.2. Behavioral Outcomes

Sleep patterns exert significant and quantifiable influence on behavioral regulation, especially through their interaction with screen time.

Guerrero et al. [17] found that increased time watching television or movies was linked to greater rule-breaking behavior by 5.9% (IRR = 1.059), social problems by 5% (IRR = 1.050), aggressive behavior by 4% (IRR = 1.040), and thought problems by 3.7% (IRR = 1.037). Playing mature-rated video games was also associated with greater somatic complaints (IRR = 1.041) and aggressive behavior (IRR = 1.039), alongside reduced sleep duration (IRR = 0.938). Importantly, longer sleep duration predicted an 8.8–16.6% decrease in problem behaviors (IRRs 0.834–0.905), mediating the relationship between screen time and behavioral symptoms.

Zink et al. [18], analyzing 10,828 youth over one year, found that girls obtaining 9–11 h of sleep per night had lower odds of withdrawn/depressed symptoms (OR = 0.6, 95% CI 0.4–0.8) and somatic complaints (OR = 0.8, 95% CI 0.6–0.97), compared to those sleeping less than 9 h. In contrast, girls with >2 h weekend screen time had higher odds of withdrawn/depressed symptoms (OR = 1.6, 95% CI 1.1–2.2); no significant associations were found in boys.

Ranum et al. [20], in a prospective cohort of 799 children followed from ages 4 to 12, found that shorter sleep duration at age 6 (β = −0.44, 95% CI −0.80 to −0.08, *p* = 0.02) and age 8 (β = −0.47, 95% CI −0.83 to −0.11, *p* = 0.01) predicted increased symptoms of emotional disorders two years later. Among boys, shorter sleep at age 8 (β = −0.65, 95% CI −1.22 to −0.08, *p* = 0.03) and age 10 (β = −0.58, 95% CI −1.07 to −0.08, *p* = 0.02) was associated with more behavioral disorder symptoms two years later; these associations were not observed in girls.

#### 3.3.3. Emotional Well-Being

The reviewed studies underscored the role of sleep in emotional regulation, with both its quality and continuity influencing affective functioning.

Cao et al. [25] reported that emotional abuse in early childhood conferred a 71% increased risk of belonging to the high-decreasing sleep score group in adolescence (incident rate ratio = 1.71, 95% CI 1.08–271). There was a dose–response relationship: experiencing more types of maltreatment in early childhood markedly increased the likelihood of poor sleep quality trajectories.

Karlovich et al. [19] found that peer victimization at baseline predicted higher levels of anxiety, depression, irritability, and poor emotion coping at the end of the school year. Sleep quality moderated the effect on emotion dysregulation: among children with high sleep quality, peer victimization (β = 0.22, *p* < 0.01) was linked to greater emotion dysregulation, compared to those with low sleep quality where dysregulation levels were moderate regardless of victimization.

Zink et al. [18] found that longer sleep duration (9–11 h/night) in girls reduced the risk of withdrawn/depressed symptoms (OR = 0.6, 95% CI 0.4–0.8) and somatic complaints (OR = 0.8, 95% CI 0.6–0.97) at one-year follow-up. However, no significant relationship was observed for anxious/depressed symptoms or for boys.

#### 3.3.4. Physical Health

The study of Zink et al. [24] found that reallocating just 30 min of daily screen time to sleep over one year was associated with significant reductions in BMI z-scores among boys (β = −0.02 to −0.03; 95% CI: −0.05 to −0.01; *p* < 0.05). Greater time spent socializing via screens was associated with higher BMI z-scores among boys (CoDA β = 0.05, 95% CI: 0.02 to 0.08), while no similar associations were found for girls. Among girls, replacing 30 min of any type of screen time with physical activity reduced BMI z-scores by β = −0.03 (95% CI: −0.05 to −0.002) for socializing, streaming, or gaming.

## 4. Discussion

A key innovation of this review is the integration of recent longitudinal and objective sleep studies with advanced computational methods, a departure from earlier syntheses that relied primarily on cross-sectional and self-reported data. This review also uniquely addresses the interplay of sleep with digital media and physical activity using emerging analytic techniques like compositional analysis. By explicitly foregrounding socioeconomic, gender, and digital context moderators, this work provides a more detailed and contemporary account of sleep’s developmental impacts than prior reviews.

The findings of this review consolidate the role of sleep as a dynamic and central component in child development. Rather than acting solely as an outcome of lifestyle or clinical conditions, sleep functions as an active regulatory mechanism that modulates core developmental domains—cognitive performance [11,36], emotional regulation [18,20], behavioral adjustment [17,21], and physical health [24].

Particularly compelling is the evidence positioning sleep as a mediator in the pathway between environmental exposures—such as screen time—and behavioral outcomes. Guerrero et al. [14] found that sleep duration mediated the relationship between screen time and problem behaviors, while Zink et al. [18] reported that sleep buffered the emotional impact of screen use, especially in girls. These findings are supported by broader literature emphasizing the regulatory role of sleep in affective and executive functioning [1,6], and suggest that interventions targeting sleep may disrupt cascading effects of digital overstimulation on child development.

In addition to mediation, moderation effects are evident across several studies. Gender differences were highlighted by Lee et al. [11], where cognitive performance gains associated with longer sleep were more pronounced in boys, and by Zink et al. [18], where improved emotional outcomes in response to sleep were stronger in girls. Socioeconomic factors also played a key role, as suggested by Souto-Manning & Melvin [13], with lower-income children facing greater risk of disrupted sleep due to adverse home environments. These interactions point to the necessity of considering how sleep interacts with contextual and biological variables in shaping developmental trajectories.

Despite these valuable insights, limitations in the current body of evidence must be acknowledged. The predominance of cross-sectional designs [10,29,30] limits causal inference, and reliance on self-report measures in studies such as Giddens et al. [32] or McGlinchey et al. [34] introduces potential biases. Few studies used objective methodologies such as actigraphy [28] or neuroimaging [10,31], and even fewer adopted longitudinal designs [22,24]. These methodological gaps hinder our ability to understand how sleep interacts dynamically with behavioral and environmental factors over time.

Moving forward, there is a critical need to embrace ecologically valid and sustainable methodologies. Studies such as Cao et al. [19] and Zhang et al. [23] illustrate the value of longitudinal approaches in capturing developmental changes in sleep and emotional outcomes. The use of wearable technologies, digital sleep diaries, and passive sensing could enable real-time monitoring of sleep behaviors in natural contexts, enhancing both precision and feasibility.

Moreover, the integration of computational tools such as machine learning can support the development of predictive models and risk profiles. For example, identifying children with high screen time, low sleep quality, and signs of emotional dysregulation—such as those described by Zink et al. [18] and Ranum et al. [21]—could inform early, personalized interventions. These approaches allow for simulation of longitudinal effects and detection of non-linear interactions often missed by traditional statistical methods.

From a public health perspective, these findings support the need to embed sleep assessment and education into pediatric and educational settings. The study by Ren et al. [35] demonstrate that even simple behavioral strategies—like consistent bedtime routines—can improve sleep and downstream functioning. These findings reinforce sleep’s potential as a modification point for early intervention, particularly in vulnerable populations disproportionately affected by environmental stressors and digital overload.

In sum, the reviewed evidence not only affirms the foundational role of sleep in child development but expands it—revealing sleep as a developmental mechanism through which risk is transmitted, and potentially mitigated.

### Limitations and Future Directions

This systematic review is subject to some limitations that should be acknowledged when interpreting its findings. First, the majority of the included studies were cross-sectional in design, which constrains the ability to draw causal inferences about the relationships between sleep, cognitive function, emotional regulation, and behavioral outcomes. Additionally, many studies relied on self-reported measures of sleep and behavior, which are vulnerable to recall bias, social desirability effects, and limited ecological validity. An important consideration is that negative self-report bias may similarly influence how both children and parents rate sleep and behavioral difficulties, leading to spurious associations driven by shared method variance rather than true links. Future research should consider incorporating measures of social desirability and other response biases to help distinguish these effects.

A second limitation relates to the selection criteria of this review. By focusing exclusively on studies involving typically developing children aged 6–12 years, the analysis excludes insights from younger children, adolescents, and clinical populations. While this enhances the homogeneity of the sample, it may also restrict the generalizability of the findings to broader developmental contexts. Furthermore, most studies originated from North America and high-income countries, limiting cross-cultural perspectives on sleep and development.

However, this review aimed to minimize traditional limitations by prioritizing papers utilizing objective sleep measures, more recent longitudinal cohorts, and digital behavioral indicators—features not systematically covered in previous syntheses.

Given these constraints, future research should prioritize the use of longitudinal designs, which are better suited to exploring temporal dynamics and causal pathways. There is also a clear need to incorporate objective and multi-informant assessment methods, such as actigraphy, wearable sensors, and passive data collection tools, to capture sleep patterns in real-life settings. These methods would provide more accurate and granular data on sleep behaviors and their fluctuations across time and context.

Future studies should adopt computational and predictive frameworks, including machine learning, to identify subgroups of children with shared risk profiles—such as high screen use, poor sleep quality, and emerging emotional difficulties. These approaches can enhance early detection and allow for personalized intervention strategies, particularly in socioeconomically vulnerable populations.

From a translational standpoint, future research should also explore how sleep-related indicators can be integrated into educational and healthcare screening tools, contributing to proactive developmental monitoring. Investigating the long-term consequences of early sleep disruption on academic achievement, mental health, and quality of life may also yield valuable insights into preventive care.

As children’s sleep environments increasingly include digital screens and variable routines, expanding the methodological and conceptual toolkit of sleep research will be essential for advancing our understanding of its role as a mediating and moderating variable in child development, and for translating that knowledge into meaningful change in policy and practice.

## 5. Conclusions

This review highlights sleep as a central and dynamic factor in child development, with consistent evidence linking sleep quality and duration to cognitive, emotional, behavioral, and physical outcomes. More than a passive background variable, sleep emerges as a modifiable mechanism that can mediate and moderate the effects of environmental exposures—such as digital screen use, adverse family conditions, and socioemotional stress.

The cumulative findings suggest that sleep disturbances may not only co-occur with developmental difficulties but may actively shape their trajectory over time. Moderators such as gender and socioeconomic status further underscore the need for more nuanced, interactionist models of development that account for individual differences in vulnerability and resilience.

Given the predominance of cross-sectional and self-reported data in current research, future studies must adopt more rigorous designs—combining longitudinal approaches, objective sleep measures, and real-time data collection through digital tools. Computational models and machine learning offer promising avenues for identifying risk profiles and simulating long-term outcomes based on sleep and behavioral patterns.

Ultimately, sleep represents a concrete and accessible point of intervention. Promoting healthy sleep habits—particularly in vulnerable populations—may help prevent emotional, behavioral, and academic difficulties before they consolidate. Embedding sleep assessment into child health monitoring and educational strategies could play a transformative role in supporting children’s developmental potential in an increasingly digital and demanding world. These findings underscore the need to integrate sleep-based screening and preventive strategies in both clinical and educational settings, with focused on those most at risk.

## Figures and Tables

**Table 1:** Quality of the methodological aspects of the primary studies (Cohort Studies).

Studies	1	2	3	4	5	6	7	8	9	10	11	Overall Score	Interpretation of the Quality Result
Cao et al. [19]	Y	Y	Y	Y	Y	Y	Y	Y	Y	Y	Y	100.00%	High
Karlovich et al. [20]	Y	Y	Y	Y	Y	N	Y	Y	Y	Y	Y	90.91%	High
Ranum et al. [21]	Y	Y	Y	Y	Y	Y	Y	Y	U	Y	Y	90.91%	High
Yang et al. [22]	Y	Y	Y	Y	Y	U	Y	Y	U	Y	Y	81.82%	High
Zang et al. [23]	Y	Y	Y	Y	Y	Y	Y	Y	Y	Y	Y	100.00%	High
Zink et al. [24]	Y	Y	Y	Y	Y	U	Y	Y	Y	Y	Y	90.91%	High
Zink et al. [18]	Y	Y	Y	Y	Y	Y	Y	Y	Y	Y	Y	100.00%	High

Note. Prepared by the author based on the JBI Critical Appraisal Checklist for cohort studies [25].The checklist includes eleven criteria evaluating methodological quality, such as group comparability, validity and reliability of exposure and outcome measures, identification and management of confounding factors, adequacy of follow-up, and appropriateness of statistical analyses. The questions were answered with the options of “Y = Yes”, “N = No”, “U = Uncertain”, or “Not Applicable”. We calculated “Yes” response percentages for each study, and the quality of each study was interpreted as a percentage as follows: High = 80% to 100%; reasonable = 50% to 79% and Low ≤ 50% [26].

**Table 2:** Quality of the methodological aspects of the primary studies (Cross-sectional Studies).

Studies	1	2	3	4	5	6	7	8	Overall Score	Interpretation of the Quality Result
Barel & Tzischinsky [27]	Y	Y	Y	Y	Y	U	Y	Y	87.50%	High
Bastien et al. [28]	Y	Y	Y	Y	U	U	Y	Y	75.00%	Reasonable
Buja et al. [29]	Y	Y	Y	Y	Y	Y	Y	Y	100.00%	High
Cheng et al. [10]	Y	Y	Y	Y	Y	Y	Y	Y	100.00%	High
Chiu et al. [30]	Y	Y	Y	Y	Y	Y	Y	Y	100.00%	High
Hehr et al. [31]	Y	Y	Y	Y	Y	Y	Y	Y	100.00%	High
Gerrero et al. [17]	Y	Y	U	Y	Y	Y	Y	Y	87.50%	High
Giddens et al. [32]	Y	Y	Y	Y	Y	Y	Y	Y	100.00%	High
Jessel et al. [33]	Y	Y	Y	U	Y	Y	U	Y	75.00%	Reasonable
McGlinchey et al. [34]	Y	Y	Y	Y	U	N	Y	Y	75.00%	Reasonable
Ren et al. [35]	Y	Y	Y	Y	Y	Y	Y	Y	100.00%	High
Yan et al. [36]	Y	Y	Y	Y	Y	Y	Y	Y	100.00%	High

Note. Prepared by the author based on the JBI Critical Appraisal Checklist for analytical cross-sectional studies [25]. The eight assessment items were used to assess the methodological aspects of each study used, with the following questions: (1) Were the criteria for inclusion in the sample clearly defined? (2) Were the study subjects and the setting described in detail? (3) Was the exposure measured in a valid and reliable way? (4) Were objective, standard criteria used for measurement of the condition? (5) Were confounding factors identified? (6) Were strategies to deal with confounding factors stated? (7) Were the outcomes measured in a valid and reliable way? (8) Was appropriate statistical analysis used? The questions were answered with the options of “Y = Yes”, “N = No”, “U = Uncertain”, or “Not Applicable”. We calculated “Yes” response percentages for each study, and the quality of each study was interpreted as a percentage as follows: High = 80% to 100%; reasonable = 50% to 79% and Low ≤ 50% [26].

**Table 3:** Study Characteristics Overview.

Reference	Title	Journal	Sample	Location	Population Focus
Bastien et al. [28]	Habitual sleep and intraindividual variability of sleep in gifted children: an actigraphy study	Journal of Clinical Sleep Medicine	62 gifted children and 62 typically developing children (~9.6 years)	Canada	Gifted children and typically developing peers
Buja et al. [29]	Is adherence to the Mediterranean diet associated with good sleep duration in primary-school children?	Frontiers in Pediatrics	267 Italian children aged 6 years	Italy	General population
Cheng et al. [10]	Sleep duration, brain structure, and psychiatric and cognitive problems in children	Molecular Psychiatry	11,067 children aged 9–11 years	United States	General population
Giddens et al. [32]	Disparities in sleep duration among American children: effects of race and ethnicity, income, age, and sex	PNAS	4207 children aged 9–13 years	United States	Racial/ethnic disparities and socioeconomic factors
Guerrero et al. [17]	Screen time and problem behaviors in children: exploring the mediating role of sleep duration	International Journal of Behavioral Nutrition and Physical Activity	11,875 children aged 9–10 years	United States	High screen time users
Hehr et al. [31]	Getting a Good Night’s Sleep: Associations Between Sleep Duration and Parent-Reported Sleep Quality on Default Mode Network Connectivity in Youth	Journal of Adolescent Health	3798 children aged 10.6–13.4 years	United States	General population
Jessel et al. [33]	Sleep Quality and Duration in Children That Consume Caffeine: Impact of Dose and Genetic Variation in ADORA2A and CYP1A	Genes—MDPI	6112 children aged 9–10 years	United States	High caffeine intake
Lee et al. [11]	Association Between Sleep Duration and Intelligence Quotient in 6-Year-Old Children	International Journal of Behavioral Medicine	538 children aged 6 years	South Korea	General population
Yan et al. [36]	Frontoparietal Response to Working Memory Load Mediates the Association between Sleep Duration and Cognitive Function in Children	Brain Sciences—MDPI	4930 children aged 9–10 years	United States	General population
Yang et al. [21]	Effects of Sleep Duration on Neurocognitive Development in U.S. Early Adolescents: A Propensity Score Matched, Longitudinal Observational Study	Lancet Child and Adolescent Health	8323 adolescents aged 9–10 years	United States	General population
Zink et al. [24]	Longitudinal Associations of Screen Time, Physical Activity, and Sleep Duration with Body Mass Index in U.S. Youth	International Journal of Behavioral Nutrition and Physical Activity	10,544 youth aged 9–11 years	United States	High screen time and physical activity patterns
Zink et al. [18]	Examining the Bidirectional Associations Between Sleep Duration, Screen Time, and Internalizing Symptoms in the ABCD Study	Journal of Adolescent Health	10,828 youth aged 9–11 years	United States	General population
Barel & Tzischinsky [27]	The Role of Sleep Patterns from Childhood to Adolescence in Vigilant Attention	International Journal of Environmental Research and Public Health	104 participants (46 children aged 6–9 years, 58 adolescents aged 13–19 years)	Northern Israel	Children and adolescents assessing sleep and vigilance differences
Cao et al. [19]	Longitudinal trajectories of sleep quality in correlation with maltreatment in early childhood	Journal of Affective Disorders	1611 early adolescents (mean age: 12.5 years, SD = 0.5)	China	Children exposed to early childhood maltreatment
Chiu et al. [30]	Higher Tablet Use Is Associated With Better Sustained Attention Performance but Poorer Sleep Quality in School-Aged Children	Frontiers in Psychology	162 children aged 6.5–8.3 years	Australia	High tablet usage and its effects
Karlovich et al. [20]	Longitudinal Associations Between Peer Victimization and Emotional Difficulties in Schoolchildren: The Role of Sleep Quality	School Mental Health	293 children aged 8–11 years (52% girls)	United States	Peer victimization and emotional regulation
McGlinchey et al. [34]	Foster Caregivers’ Perceptions of Children’s Sleep Patterns, Problems, and Environments	Journal of Pediatric Psychology	485 foster caregivers of children aged 4–11 years	United States	Foster care children
Zhang et al. [23]	Latent Profiles of Sleep Patterns in Early Adolescence: Associations With Behavioral Health Risk	Journal of Adolescent Health	3326 early adolescents aged 10.58–13.67 years	United States	Adolescents with varying sleep profiles and behavioral health risks
Ranum et al. [21]	Association Between Objectively Measured Sleep Duration and Symptoms of Psychiatric Disorders in Middle Childhood	JAMA Network Open	799 children aged 6–12 years	Norway	General population
Ren et al. [35]	The Relative Importance of Sleep Duration and Bedtime Routines for the Social-Emotional Functioning of Chinese Children	Journal of Developmental and Behavioral Pediatrics	228 Chinese school-aged children	China	General population

**Table 4:** Study Objectives and Key Findings.

Reference	Objective	Study Design	Outcome Measures	Results	Limitations
Bastien et al. [28]	Investigate habitual sleep, night-to-night sleep variability, and parental reports of sleep in gifted versus typically developing children.	Observational cross-sectional study	Actigraphy to assess sleep duration, sleep efficiency, and night-to-night variability; parental reports via the Children’s Sleep Habits Questionnaire.	Gifted children exhibited lower sleep efficiency (mean difference ≈ 5%), more wake time after sleep onset (WASO), and greater night-to-night sleep variability compared to typically developing peers (*p* < 0.05). They showed less social jetlag but had more clinically significant parent-reported sleep problems.	Small sample size; focus on a specific population.
Buja et al. [29]	Examine the association between adherence to the Mediterranean diet and sleep duration in primary-school children.	Cross-sectional observational study	Parent-reported sleep duration and diet adherence measured by the Mediterranean Diet Quality Index	Longer sleep duration was positively associated with higher adherence to the Mediterranean diet (OR = 1.15 per additional hour of sleep, 95% CI: 1.05–1.26, *p* = 0.003). Children sleeping > 9 h had 20% higher odds of better diet adherence than those sleeping < 8 h	Self-reported data may introduce bias; cross-sectional design limits causal inference.
Cheng et al. [10]	Explore the relationship between sleep duration, brain structure, cognitive performance, and psychiatric problems in children.	Cross-sectional observational study	Brain morphometry, sleep duration reported by parents, psychiatric symptom assessment.	Shorter sleep duration was associated with greater psychiatric symptoms in children (β = −0.22, *p* < 0.001) and their parents, as well as structural changes in brain regions involved in cognition and emotion regulation (e.g., thinner prefrontal cortex). Cognitive performance was positively related to sleep duration (β = 0.24, *p* < 0.01).	Large dataset but cross-sectional design limits causality; sleep duration based on parent report; inability to account for all confounders.
Giddens et al. [32]	Investigate disparities in sleep duration among American children by race, ethnicity, income, age, and sex.	Cross-sectional observational study	Weekly actigraphy (Fitbit), demographic and socioeconomic information.	Black children slept, on average, 34 min less per night than White children (Cohen’s d = 0.95); the disparity reached 41 min between Black children from low-income homes and White children from high-income homes (Cohen’s d = 1.15). Children from low-income families slept 16 min less compared to higher-income peers (Cohen’s d = 0.44). Boys slept 7 min less than girls (Cohen’s d = 0.18). Older children and those with higher BMI also had shorter sleep durations. The disparities were primarily explained by later bedtimes in Black and low-income children, not by school start time. Neighborhood deprivation, parent’s age at child’s birth, and discrimination did not significantly contribute to sleep time.	Overrepresentation of White children and those from high-income households; use of commercial Fitbit with closed algorithm; cross-sectional design precludes causality.
Guerrero et al. [17]	Assess the association between screen time, problem behaviors, and the mediating role of sleep duration.	Cross-sectional observational study	Parent-reported screen time, sleep duration, and problem behaviors assessed through validated questionnaires.	Higher screen time was associated with more behavioral problems, partially mediated by shorter sleep duration (indirect effect = 0.04, *p* < 0.05). Sleep duration accounted for approximately 20% of the total effect of screen time on problem behaviors. Direct and indirect effects remained significant after controlling for covariates.	Potential unmeasured confounders; cross-sectional design; reliance on parent reports for all variables.
Hehr et al. [31]	Investigate associations between sleep duration, parent-reported sleep quality, and resting-state functional connectivity (rs-FC) in core neurocognitive brain networks in youth.	Cross-sectional analysis	Sleep duration and wake after sleep onset (WASO) via Fitbit; parent-reported disturbances; resting-state functional connectivity (rs-FC) of default mode network (DMN) and anticorrelated networks (dorsal attention network (DAN), frontoparietal, salience)	Shorter sleep duration associated with weaker within-DMN connectivity (β unspecified) and weaker anti-correlation between DMN-DAN (*p* < 0.05) and DMN-frontoparietal networks (*p* < 0.05). Greater WASO correlated with altered DMN-DAN connectivity, especially in those with lower total sleep hours. These alterations suggest increased risk for emotional and attentional vulnerabilities	Generalizability limited by sample demographics.
Jessel et al. [33]	Examine the effects of daily caffeine dose and genetic variations in ADORA2A and CYP1A on sleep quality and duration in children.	Cross-sectional observational study	Sleep quality and duration assessed via parent reports; caffeine intake from various beverages estimated by questionnaire; genotyping for ADORA2A rs5751876 and CYP1A variants.	Higher daily caffeine doses were associated with significantly lower odds of children reporting more than 9 h of sleep per night (OR = 0.81, 95% CI = 0.74–0.88, *p* = 1.2 × 10^−6^). For each mg/kg/day increase in caffeine, odds of sleeping >9 h decreased by 19% (95% CI = 12–26%). No significant associations were found between genetic variants and sleep quality, duration, or caffeine dose, nor were gene-by-caffeine dose interactions observed.	Cross-sectional design precludes causal inferences; reliance on parent-reported sleep and self-reported caffeine intake; genetic moderation effects may require larger samples or different age groups for detection.
Lee et al. [11]	Investigate the effects of sleep duration on IQ in 6-year-old children, focusing on sex differences.	Cross-sectional observational study	Parent-reported sleep duration; IQ assessed using standardized intelligence tests.	Longer sleep duration was associated with higher IQ scores (β = 0.18, *p* < 0.05). The association was significant in boys (β = 0.25, *p* < 0.01) but not in girls (β = 0.12, *p* = 0.09), indicating sex-specific effects of sleep duration on IQ.	Single-country study; self-reported sleep duration.
Yan et al. [36]	Explore neural mechanisms underlying the association between sleep duration and cognitive function, focusing on frontoparietal activation.	Cross-sectional observational study	Parent-reported sleep duration; cognitive function assessed with working memory tasks; functional MRI measuring frontoparietal brain activation during tasks	Longer sleep duration was positively associated with better cognitive performance (β = 0.22, *p* < 0.01). Frontoparietal activation mediated this effect, with the mediation effect stronger in girls than boys (indirect effect β = 0.10 vs. 0.05). Sex differences suggest differential neural pathways involved.	Cross-sectional design; limited control of confounding factors.
Yang et al. [22]	Investigate long-term effects of insufficient sleep on neurocognitive development, behavioral, cognitive, and brain outcomes.	Longitudinal observational study	Sleep duration (self/parent-report), behavioral/mental health (CBCL, cognition tests), brain structure/function (MRI, rs-fMRI)	Insufficient sleep led to higher behavioral problems (Cohen’s d = 0.17), cognitive deficits (d = 0.08), stable rs-fMRI/structural deficits over 2 yrs (r = 0.54, r = 0.52), all *p* < 0.0001	Self-reported sleep data; potential confounders.
Zink et al. [24]	Examine the longitudinal associations between screen time, physical activity, sleep duration, and BMI.	Longitudinal observational study	Self-reported screen time, accelerometer-measured physical activity and sleep duration; clinical BMI z-scores.	Physical activity was inversely associated with BMI in females (β = −0.20, *p* < 0.01), while longer sleep duration was inversely associated with BMI in males (β = −0.15, *p* < 0.05). Screen time showed no direct effect on BMI but was indirectly linked through sleep duration and physical activity. Sex-specific differences suggest different behavioral targets for obesity prevention.	One-year follow-up; reliance on self-reported data.
Zink et al. [18]	Explore bidirectional associations between sleep duration, screen time, and internalizing symptoms, focusing on gender differences.	Longitudinal observational study	Parent-reported sleep duration, self-reported screen time (weekdays/weekends), CBCL internalizing subscales (withdrawn/depressed, anxious/depressed, somatic).	In females, 9–11 h sleep (vs <9 h) reduced risk for withdrawn/depressed (OR = 0.6, 95% CI 0.4–0.8, *p* < 0.001) and somatic complaints (OR = 0.8, 95% CI 0.6–0.97, *p* = 0.021) after 1 yr; >2 h weekend screen time raised risk for withdrawn/depressed (OR = 1.6, CI 1.1–2.2, *p* = 0.013); No significant associations in males or for anxious/depressed symptoms.	Short follow-up; reliance on caregiver-reported symptoms.
Barel & Tzischinsky [27]	Examine the role of sleep patterns in vigilant attention performance among children and adolescents.	Cross-sectional observational study	Sleep duration, efficiency, variability, and vigilant attention (PVT-B measures).	Adolescents outperformed children in vigilant attention tasks despite shorter sleep duration. Weekday sleep loss negatively impacted cognitive vigilance in both groups, with adolescents showing greater sensitivity (r = −0.45, *p* < 0.01) to sleep loss. Higher sleep variability was associated with worse attention (β = −0.39, *p* < 0.05).	Limited generalizability due to the small sample size and geographic specificity.
Cao et al. [19]	Examine the relationship between early childhood maltreatment and sleep quality trajectories in adolescents.	Longitudinal cohort study	Self-reported early childhood maltreatment (emotional and physical abuse) and repeated sleep quality assessments, analyzed using group-based trajectory modeling	Four distinct sleep quality trajectories were identified: low (25%), moderate-low (51%), moderate-increasing (17%), and high-decreasing (7%). Emotional abuse was associated with a 71% increased risk of belonging to the high-decreasing trajectory group (Incidence Rate Ratio (IRR) = 1.71, 95% CI: 1.08–2.71). Physical abuse also increased risk for poorer sleep trajectories. A dose–response relationship existed between number of maltreatment types experienced and risk of poor sleep trajectory.	Relied on self-reported data, which may introduce reporting bias.
Chiu et al. [30]	Investigate the associations between screen use, sustained attention, and sleep quality in school-aged children.	Cross-sectional observational study	Screen time (tablet, TV, smartphone) and sustained attention; sleep quality and duration.	Higher tablet use was associated with better sustained attention scores (β = 0.15, *p* < 0.05), but poorer sleep quality (β = −0.20, *p* < 0.01). Sleep duration was positively correlated with sustained attention (r = 0.18, *p* < 0.05). Effects of sleep and screen time on attention were independent in multivariate models controlling for age, sex, and socioeconomic status.	Cross-sectional design limits causal inference. Self-reported screen time and sleep measures may introduce bias.
Karlovich et al. [20]	Explore bidirectional associations between peer victimization, emotional difficulties, and moderating effects of sleep.	Longitudinal observational study	Peer victimization, emotional difficulties (e.g., anxiety, depression) assessed via standardized questionnaires, and sleep quality, assessed through self-report measures.	Peer victimization predicted increased emotional difficulties over time, but emotional difficulties did not predict later victimization (β = 0.34, *p* < 0.01). Sleep quality significantly moderated the effect of victimization on emotional dysregulation; children with poorer sleep showed stronger associations between victimization and emotional problems (interaction term β = −0.22, *p* < 0.05).	Small sample size and reliance on self-reported measures.
McGlinchey et al. [34]	Examine caregivers’ perceptions of foster children’s sleep patterns and problems.	Cross-sectional observational study	Caregiver-reported sleep issues including sleep latency, night awakenings, nightmares, and snoring; sleep environments assessed via caregiver interviews.	Common problems included prolonged sleep latency(>30 min), awakenings, nightmares, and reliance on caregivers. Trauma history and environmental factors influenced sleep patterns. Foster children exhibited higher rates of sleep disturbances compared to general population estimates.	Limited to foster children; limited generalizability; reliance on caregiver report rather than objective sleep measures
Zhang et al. [23]	Identify distinct sleep patterns in adolescents and examine associations with behavioral health risks.	Longitudinal cohort study	Sleep patterns measured by Fitbit (duration, efficiency, latency); behavioral outcomes assessed via standardized psychological questionnaires.	Four distinct sleep profiles emerged: stable good sleepers (40%), moderate poor sleepers (30%), poor irregular sleepers (20%), and declining sleepers (10%). Poor irregular and declining sleepers showed significantly higher rates of behavioral problems, including attentional difficulties (β = 0.35, *p* < 0.01) and rule-breaking behaviors (β = 0.29, *p* < 0.05) compared to stable good sleepers.	Limited to Fitbit-measured sleep data; does not account for other health-related factors.
Ranum et al. [21]	Investigate the prospective associations between sleep duration and symptoms of emotional and behavioral disorders at ages 6–12 years.	Longitudinal cohort study	Sleep duration assessed via accelerometer; psychiatric symptoms assessed via clinical interviews.	Shorter sleep duration predicted increased symptoms of emotional disorders in both genders and behavioral disorders in boys over follow-up (β ≈ −0.3, *p* < 0.01). No evidence of reverse causality was observed.	Limited generalizability due to a Norwegian-only sample and potential residual confounding despite rigorous controls.
Ren et al. [35]	Investigate the unique contributions of sleep duration and bedtime routines to social-emotional functioning of children.	Cross-sectional observational study	Parent-reported sleep duration, consistency of bedtime routines, and social-emotional functioning assessed via standardized behavioral rating scales.	Longer sleep duration was associated with fewer behavioral problems (β = −0.27, *p* < 0.001), while consistent bedtime routines contributed to better social skills (β = 0.33, *p* < 0.001). Significant sex differences were observed: bedtime routine consistency was more strongly linked to social skills in boys, while sleep duration had a stronger association in girls.	Cross-sectional design limits causal inference; reliance on parent reports may introduce response bias; potential cultural specificity limiting generalizability.

## Data Availability

The original contributions presented in this study are included in the article/Supplementary Material. Further inquiries can be directed to the corresponding author.

## Supplementary Materials

The following supporting information can be downloaded at: https://www.mdpi.com/article/10.3390/clockssleep7040066/s1.
