# Correction: Félix, A.; Candeias, A. Sleep as a Developmental Process: A Systematic Review of Cognitive, Emotional, and Behavioral Outcomes in Children Aged 6–12 Years. *Clocks & Sleep* 2025, *7*, 66

**DOI:** 10.3390/clockssleep8010010

**Published:** 2026-02-27

**Authors:** Adriana Félix, Adelinda Candeias

**Affiliations:** School of Health and Human Development, Comprehensive Health Research Centre, Universidade de Évora, 7000-812 Évora, Portugal; d60733@alunos.uevora.pt

## Missing Funding

In the original publication [1], the funder Fundação para a Ciência e Tecnologia (FCT), I.P., under the project UID/06291/2025, was not included. The Correct Funding statement should read:

**Funding:** This work was funded by national funds through FCT—Fundação para a Ciência e Tecnologia, I.P., in the framework of the Comprehensive Health Research Centre (CHRC), under the project UID/06291/2025. The funders had no role in the design of the study; in the collection, analysis, or interpretation of data; or in the writing of the manuscript.
**Acknowledgments:** The authors would like to acknowledge the University of Évora for its institutional support and the Comprehensive Health Research Centre (CHRC) for providing the scientific and infrastructural framework that supported the development of this work.
  The authors state that the scientific conclusions are unaffected. This correction was approved by the Academic Editor. The original publication has also been updated.
