# Supplementary material for: Sleep as a Developmental Process: A Systematic Review of Cognitive, Emotional, and Behavioral Outcomes in Children Aged 6–12 Years
Source: Clocks Sleep. 2025 Nov 14;7(4):66. doi: 10.3390/clockssleep7040066 (PMC12641626; doi:10.3390/clockssleep7040066)
Supplement: Supplementary file 1 [file clockssleep-07-00066-s001.zip › clockssleep-3918856-supplementary.pdf]

# Supplementary Material 1: Full Database Search Strategies

This appendix provides the full search strategies employed in each database for the systematic review, as per PRISMA 2020 guidelines.

**Date of search**: December 13, 2024

**Timeframe covered**: Publications from 2019 through 2024

**Language**: English

**Document type**: Peer-reviewed articles (where available)

**Databases:** EBSCO, Scopus, Web of Science

## EBSCO

**Fields Searched**: Title (TI), Subject Terms (SU)

**Search String**:

TI (sleep patterns OR sleep quality OR sleep duration) AND SU (neurodevelopment OR cognitive development OR emotional development) AND (children OR childhood)

**Limits applied**:

- Publication Years: 2019–2024
- Language: English

## Scopus

**Fields Searched**: Title, Abstract, Keywords

**Search String:**

TITLE-ABS-KEY (sleep AND patterns OR sleep AND quality OR sleep AND duration) AND TITLE-ABS-KEY (neurodevelopment OR cognitive AND

development OR emotional AND development) AND TITLE-ABS-KEY (children OR childhood) AND PUBYEAR > 2018 AND PUBYEAR < 2025 AND LIMIT-TO (LANGUAGE, "English") AND LIMIT-TO (PUBSTAGE, "final")

Limits applied:

- Publication Years: 2019–2024
- Language: English
- PubStage: Final

Web of Science

Fields Searched:

- In "Title": sleep patterns OR sleep quality OR sleep duration
- In "Topic": neurodevelopment OR cognitive development OR emotional development
- In "Topic": children OR childhood

Search String:

(sleep patterns OR sleep quality OR sleep duration) (Title) AND (neurodevelopment OR cognitive development OR emotional development) (Topic) AND (children OR childhood) (Topic) AND (2019 OR 2020 OR 2021 OR 2022 OR 2023 OR 2024) (Publication Years) AND English (Languages)

Limits applied:

- Publication Years: 2019–2024
- Language: English

Search Strategy Notes

- Queries were adapted to the unique syntax and field functionality of each database.
- All results were exported and deduplicated before screening.
- No manual exclusions or additional filters were applied beyond those listed.
